# WONDER-01: immediate necrosectomy vs. drainage-oriented step-up approach after endoscopic ultrasound-guided drainage of walled-off necrosis—study protocol for a multicentre randomised controlled trial

**DOI:** 10.1186/s13063-023-07377-y

**Published:** 2023-05-24

**Authors:** Tatsuya Sato, Tomotaka Saito, Mamoru Takenaka, Takuji Iwashita, Hideyuki Shiomi, Toshio Fujisawa, Nobuhiko Hayashi, Keisuke Iwata, Akinori Maruta, Tsuyoshi Mukai, Atsuhiro Masuda, Saburo Matsubara, Tsuyoshi Hamada, Tadahisa Inoue, Hiroshi Ohyama, Masaki Kuwatani, Hideki Kamada, Shinichi Hashimoto, Toshiyasu Shiratori, Reiko Yamada, Hirofumi Kogure, Takeshi Ogura, Kazunari Nakahara, Shinpei Doi, Kenji Chinen, Hiroyuki Isayama, Ichiro Yasuda, Yousuke Nakai, for the WONDERFUL study group in Japan, collaborators

**Affiliations:** 1 Department of Gastroenterology, Graduate School of Medicine, The University of Tokyo, Tokyo, Japan; 2 Department of Gastroenterology and Hepatology, Faculty of Medicine, Kindai University, Osaka, Japan; 3 First Department of Internal Medicine, Gifu University Hospital, Gifu, Japan; 4 Division of Gastroenterology and Hepatobiliary and Pancreatic Diseases, Department of Internal Medicine, Hyogo Medical University, Hyogo, Japan; 5 Department of Gastroenterology, Graduate School of Medicine, Juntendo University, Tokyo, Japan; 6 Third Department of Internal Medicine, University of Toyama, Toyama, Japan; 7 Department of Gastroenterology, Gifu Municipal Hospital, Gifu, Japan; 8 Department of Gastroenterology, Gifu Prefectural General Medical Center, Gifu, Japan; 9 Department of Gastroenterological Endoscopy, Kanazawa Medical University, Ishikawa, Japan; 10 Division of Gastroenterology, Department of Internal Medicine, Kobe University Graduate School of Medicine, Hyogo, Japan; 11 Department of Gastroenterology and Hepatology, Saitama Medical Center, Saitama Medical University, Saitama, Japan; 12 Department of Hepato-Biliary-Pancreatic Medicine, The Cancer Institute Hospital of Japanese Foundation for Cancer Research, Tokyo, Japan; 13 Department of Gastroenterology, Aichi Medical University, Aichi, Japan; 14 Department of Gastroenterology, Graduate School of Medicine, Chiba University, Chiba, Japan; 15 Department of Gastroenterology and Hepatology, Hokkaido University Hospital, Hokkaido, Japan; 16 Department of Gastroenterology and Neurology, Kagawa University, Kagawa, Japan; 17 Digestive and Life-Style Diseases, Kagoshima University Graduate School of Medicine and Dental Sciences, Kagoshima, Japan; 18 Department of Gastroenterology, Kameda Medical Center, Chiba, Japan; 19 Department of Gastroenterology and Hepatology, Mie University Hospital, Mie, Japan; 20 Division of Gastroenterology and Hepatology, Department of Medicine, Nihon University School of Medicine, Tokyo, Japan; 21 Endoscopy Center, Osaka Medical and Pharmaceutical University Hospital, Osaka, Japan; 22 Department of Gastroenterology, St. Marianna University School of Medicine, Kanagawa, Japan; 23 Department of Gastroenterology, Teikyo University Mizonokuchi Hospital, Kanagawa, Japan; 24 Department of Gastroenterology, Yuuai Medical Center, Okinawa, Japan; 25 Department of Endoscopy and Endoscopic Surgery, The University of Tokyo Hospital, Tokyo, Japan

**Keywords:** Acute necrotizing pancreatitis, Drainage, Endoscopy, Endosonography, Randomised clinical trial, Stents, Ultrasonography, Walled-off necrosis

## Abstract

**Background:**

With the increasing popularity of endoscopic ultrasound (EUS)-guided transmural interventions, walled-off necrosis (WON) of the pancreas is increasingly managed via non-surgical endoscopic interventions. However, there has been an ongoing debate over the appropriate treatment strategy following the initial EUS-guided drainage. Direct endoscopic necrosectomy (DEN) removes intracavity necrotic tissue, potentially facilitating early resolution of the WON, but may associate with a high rate of adverse events. Given the increasing safety of DEN, we hypothesised that immediate DEN following EUS-guided drainage of WON might shorten the time to WON resolution compared to the drainage-oriented step-up approach.

**Methods:**

The WONDER-01 trial is a multicentre, open-label, superiority, randomised controlled trial, which will enrol WON patients aged ≥ 18 years requiring EUS-guided treatment in 23 centres in Japan. This trial plans to enrol 70 patients who will be randomised at a 1:1 ratio to receive either the immediate DEN or drainage-oriented step-up approach (35 patients per arm). In the immediate DEN group, DEN will be initiated during (or within 72 h of) the EUS-guided drainage session. In the step-up approach group, drainage-based step-up treatment with on-demand DEN will be considered after 72–96 h observation. The primary endpoint is time to clinical success, which is defined as a decrease in a WON size to ≤ 3 cm and an improvement of inflammatory markers (i.e. body temperature, white blood cell count, and C-reactive protein). Secondary endpoints include technical success, adverse events including mortality, and recurrence of the WON.

**Discussion:**

The WONDER-01 trial will investigate the efficacy and safety of immediate DEN compared to the step-up approach for WON patients receiving EUS-guided treatment. The findings will help us to establish new treatment standards for patients with symptomatic WON.

**Trial registration:**

ClinicalTrials.gov NCT05451901, registered on 11 July 2022. UMIN000048310, registered on 7 July 2022. jRCT1032220055, registered on 1 May 2022.

## Administrative information

Note: the numbers in curly brackets in this protocol refer to SPIRIT checklist item numbers. The order of the items has been modified to group similar items (see http://www.equator-network.org/reporting-guidelines/spirit-2013-statement-defining-standard-protocol-items-for-clinical-trials/).

**Table:** 

Title {1}	WONDER-01: immediate necrosectomy vs. drainage-oriented step-up approach after endoscopic ultrasound-guided drainage of walled-off necrosis
Trial registration {2a and 2b}.	ClinicalTrials.gov NCT05451901, registered on 11 July 2022. UMIN000048310, registered on 7 July 2022. jRCT1032220055, registered on 1 May 2022.
Protocol version {3}	Version 6.0, last updated on 28 September 2022.
Funding {4}	This work was supported by grants from the Japanese Foundation for Research and Promotion of Endoscopy (#1015 to T.Sai and a multicentre research grant to Y.N.). T.H. was supported by Japan Society for the Promotion of Science (JSPS) KAKENHI grants (JP19K08362 and JP22H02841) and a grant from Takeda Science Foundation. The funders had no role in study design, data collection and analysis, decision to publish, or preparation of the manuscript.
Author details {5a}	^1^Department of Gastroenterology, Graduate School of Medicine, The University of Tokyo, 7–3-1, Hongo Bunkyo-ku, Tokyo, Japan. ^2^Department of Gastroenterology and Hepatology, Faculty of Medicine, Kindai University, Osaka, Japan. ^3^First Department of Internal Medicine, Gifu University Hospital, Gifu, Japan. ^4^Division of Gastroenterology and Hepatobiliary and Pancreatic Diseases, Department of Internal Medicine, Hyogo Medical University, Hyogo, Japan. ^5^Department of Gastroenterology, Graduate School of Medicine, Juntendo University, Tokyo, Japan. ^6^Third Department of Internal Medicine, University of Toyama, Toyama, Japan. ^7^Department of Gastroenterology, Gifu Municipal Hospital, Gifu, Japan. ^8^Department of Gastroenterology, Gifu Prefectural General Medical Center, Gifu, Japan. ^9^Department of Gastroenterological Endoscopy, Kanazawa Medical University, Ishikawa, Japan. ^10^Division of Gastroenterology, Department of Internal Medicine, Kobe University Graduate School of Medicine, Hyogo, Japan. ^11^Department of Gastroenterology and Hepatology, Saitama Medical Center, Saitama Medical University, Saitama, Japan. ^12^Department of Hepato-Biliary-Pancreatic Medicine, The Cancer Institute Hospital of Japanese Foundation for Cancer Research, Tokyo, Japan. ^13^Department of Gastroenterology, Aichi Medical University, Aichi, Japan. ^14^Department of Gastroenterology, Graduate School of Medicine, Chiba University, Chiba, Japan. ^15^Department of Gastroenterology and Hepatology, Hokkaido University Hospital, Hokkaido, Japan. ^16^Gastroenterology and Neurology, Kagawa University, Kagawa, Japan. ^17^Digestive and Life-style Diseases, Kagoshima University Graduate School of Medicine and Dental Sciences, Kagoshima, Japan. ^18^Department of Gastroenterology, Kameda Medical Center, Chiba, Japan. ^19^Department of Gastroenterology and Hepatology, Mie University Hospital, Mie, Japan. ^20^Division of Gastroenterology and Hepatology, Department of Medicine, Nihon University School of Medicine, Tokyo, Japan. ^21^ Endoscopy Center, Osaka Medical and Pharmaceutical University Hospital, Osaka, Japan. ^22^Department of Gastroenterology, St. Marianna University School of Medicine, Kanagawa, Japan. ^23^Department of Gastroenterology, Teikyo University Mizonokuchi Hospital, Kanagawa, Japan. ^24^Department of Gastroenterology, Yuuai Medical Center, Okinawa, Japan. ^25^Department of Endoscopy and Endoscopic Surgery, The University of Tokyo Hospital, Tokyo, Japan.
Name and contact information for the trial sponsor {5b}	There is no sponsor for this trial.
Role of sponsor {5c}	n/a because there is no sponsor for this trial.

## Introduction

### Background and rationale {6a}

Pancreatic fluid collections develop as late complications of severe acute pancreatitis [1]. According to the revised Atlanta classification [2], walled-off necrosis (WON) has been defined as a collection characterised by encapsulated necrosis that is observed after four weeks of the onset of acute pancreatitis. Infected WON generally results in high morbidity and mortality, and it is mandatory to manage WON appropriately to improve clinical outcomes of patients with acute pancreatitis [3–6]. Endoscopic ultrasound (EUS)-guided drainage has become a first-line treatment modality for infected WON [7]. For patients who are refractory to EUS-guided drainage, direct endoscopic necrosectomy (DEN) is a treatment option to facilitate direct removal of infected necrotic tissue within the WON and thereby, control the infection [8]. Lumen-apposing metal stents (LAMSs) have emerged as a promising treatment modality in this setting and have increased the popularity of the non-surgical treatment through serving as a transluminal port for safe and effective DEN [9–12]. However, there is a controversy over the appropriate timing of starting DEN following EUS-guided drainage of WON [13–16]. Due to potentially lethal adverse events of DEN, such as bleeding, perforation, and peritonitis [8, 17, 18], DEN is usually initiated after several days of observation with unsuccessful clinical improvement (so-called the step-up approach). Given the adverse events related to DEN, many endoscopists may select the step-up approach consisting of intense drainage procedures (rather than DEN) including additional EUS-guided drainage through another route or addition of stents/catheters (termed “drainage-oriented step-up approach”) [19, 20]. However, prolonged duration of LAMS placement may result in stent-related adverse events (e.g. bleeding, buried stent, stent occlusion with or without fever) [21–24]. Recently, with increasing technical safety of DEN according to accumulated endoscopists’ knowledge and skills, studies suggest that DEN immediately after EUS-guided drainage potentially shortens treatment duration without a substantial increase in adverse events [14]. Given these lines of evidence, we hypothesised that immediate DEN following EUS-guided drainage of WON might shorten time to WON resolution compared to the drainage-oriented step-up approach.

To test our hypothesis, we were motivated to conduct a multicentre randomised controlled trial (RCT) to investigate the superiority of immediate DEN over the drainage-oriented step-up approach in terms of time to clinical success (WON resolution). Our data would help to establish a new treatment paradigm for WON patients receiving EUS-guided treatment and to improve clinical outcomes of patients with acute pancreatitis overall.

### Objectives {7}

The primary objective of the WONDER-01 trial is to evaluate the superiority of immediate DEN over drainage-oriented step-up approach following EUS-guided drainage of WON in terms of time to clinical success. The secondary objectives include assessments of a technical success rate, procedure-related adverse events, and long-term outcomes (detailed in Table 1).

**Table 1:** The primary and secondary endpoints of the WONDER-01 trial

Primary endpoint	Secondary endpoints
Time from randomisation to clinical success^a^	Incidence of procedure-related adverse events graded by the AGREE classification [25] as well as the ASGE lexicon guideline [26]
Clinical success is defined as (1) a decrease in a WON size to 3 cm or less and (2) an improvement of at least two out of the three inflammatory markers (i.e. body temperature, white blood cell count, and C-reactive protein)	Mortality from any cause
	Technical success rate of the initial EUS-guided drainage
	Incidence of a biliary stricture and gastrointestinal obstruction
	Number of interventions
	Total procedure time
	Time requiring endoscopic and/or percutaneous drainage
	Length of hospitalisation
	Length of ICU stay
	Duration of antibiotics administration
	Total costs of interventions and hospitalisation
	Incidence of WON recurrence
	Time from clinical success to WON recurrence
	Incidence of a pancreatic pseudocyst
	Incidence of new-onset diabetes
	Incidence of clinical symptoms associated with pancreatic exocrine insufficiency
	Incidence of pancreatic cancer
	Incidence of sarcopenia
	Changes in the morphology and volume of the pancreas

^a^Patients who do not undergo clinical success within 6 months of the randomisation will be treated as cases with clinical failure

Abbreviations: *ASGE* American Society for Gastrointestinal Endoscopy, *EUS* Endoscopic ultrasound; *ICU*, Intensive care unit, *WON* Walled-off necrosis

### Trial design {8}

The WONDER-01 trial is designed as a multicentre, open-label, parallel-group, randomised controlled trial that evaluates the superiority of immediate DEN over drainage-oriented step-up approach in terms of time to clinical success among patients receiving EUS-guided drainage of WON. Patients diagnosed with symptomatic WON will be screened for the inclusion and exclusion criteria. Eligible patients will be randomised at a 1:1 ratio to either the immediate DEN group or the step-up approach group.

The WONDER-01 trial has been designed and will be implemented by the WONDERFUL (WON anD pERipancreatic FlUid coLlection) study group, which consisted of expert endoscopists, gastroenterologists, interventional radiologists, and epidemiologists at high-volume centres in Japan [27, 28].

## Methods: participants, interventions and outcomes

### Study setting {9}

The WONDER-01 trial will be conducted in tertiary care centres in Japan. Therefore, data will be collected and analysed in Japan.

### Eligibility criteria {10}

The inclusion and exclusion criteria for patient eligibility are listed in Table 2. Eligible patients must meet all inclusion criteria and none of the exclusion criteria for enrolment.

**Table 2:** Eligibility criteria for the WONDER-01 trial

Inclusion criteria	Exclusion criteria
Patients with WON defined by the revised Atlanta classification [2]	WON inaccessible via the EUS-guided approach
The longest diameter of WON is 4 cm or larger	A LAMS in situ
Patients requiring drainage for WON	Coagulopathy (platelet count < 50,000/mm^3^ or PT-INR > 1.5)
Patients with symptoms due to WON (e.g. infection, GI symptoms, or jaundice)	Antithrombotic agents cannot be discontinued according to the JGES guideline [29]
Patients aged 18 years or older	Patients who do not tolerate endoscopic procedures
Patients or their representatives provide informed consent	Pregnant women
	Patients considered inappropriate for inclusion by the investigators

Abbreviations: *EUS* Endoscopic ultrasound, *JGES* Japan Gastroenterological Endoscopy Society, *LAMS* Lumen-apposing metal stent, *PT-INR*, Prothrombin time international normalised ratio, *WON* Walled-off necrosis

Endoscopists at the participating centres (the study investigators) will perform interventions for both groups.

### Who will take informed consent? {26a}

The study investigators will obtain written informed consent from potential trial participants or authorised surrogates using the latest version of the approved consent form.

### Additional consent provisions for collection and use of participant data and biological specimens {26b}

n/a. The study data will be used for secondary purposes in future studies only after the additional approval at the institutional review board. A chance for informed consent or opt-out, as appropriate, will be provided for participants. The current study will not use biospecimens from the participants as the study protocol.

## Interventions

### Explanation for the choice of comparators {6b}

In the WONDER-01 trial, the experimental intervention is immediate DEN following EUS-guided drainage of WON, and the control intervention is a drainage-oriented step-up approach. Based on accumulating evidence [30–32], the step-up approach is currently taken as a first-line treatment option of endoscopic treatment of pancreatic fluid collections (PFCs) at many centres with an expectation of avoiding adverse events due to unnecessary DEN procedures. Given potential adverse events associated with DEN, endoscopists may postpone DEN until patients become unamenable to any non-surgical drainage procedures including percutaneous interventions (termed “drainage-oriented step-up approach”). In the drainage-oriented step-up approach group in the present trial, adjunctive treatment following the initial EUS-guided drainage is based on additional non-surgical drainage procedures (including but not limited to relocation of stents and catheters, and the multigateway/multimodality strategies), and DEN is initiated on demand after unsuccessful intensive drainage.

### Intervention description {11a}

In both experimental and control groups, EUS-guided drainage of WON and adjunctive interventions including DEN will be performed on an inpatient basis. EUS-guided drainage is conducted in a standard fashion within 72 h of the randomisation. A linear echoendoscope is advanced to the stomach or duodenum with moderate sedation, and the WON is visualised and punctured under the endosonographic guidance. To reduce the risk of air emboli, carbon dioxide insufflation will be used wherever available. A LAMS (Hot AXIOS system; Boston Scientific Japan, Tokyo, Japan) is used as the first choice, but plastic stent(s) can be used as an alternative (e.g. in case of difficulties in deploying the LAMS due to the highly solid contents). Prophylactic antibiotics will be administered. The number and diameter (15 mm or 20 mm for a LAMS; and 7 to 10 Fr for a plastic stent) of stents, the additional placement of a nasocystic catheter during the procedure, and the periprocedural use of proton pump inhibitors will be determined on the endoscopist’s discretion. A subsequent treatment sequence differs by the allocated groups as follows.

#### Experimental intervention: immediate necrosectomy following EUS-guided drainage of WON

In the immediate necrosectomy group, DEN will be initiated during the same session of the initial EUS-guided drainage or at least within 72 h of the drainage. DEN will be performed using a gastroscope, and the devices (e.g. biopsy forceps, snare, or basket catheter) used during the DEN are determined on the endoscopist’s discretion. The DEN procedures will be repeatedly performed until clinical success.

#### Control intervention: drainage-oriented step-up approach following EUS-guided drainage of WON

In the step-up approach group, an indication of additional interventions will be considered after 72–96 h observation following the initial EUS-guided drainage. In cases without sufficient clinical improvement after 72–96 h, drainage-based step-up treatment will be performed (e.g. replacement of a stent, addition of a stent and/or catheter, EUS-guided drainage [so-called multigateway technique], and/or percutaneous drainage [so-called multimodality technique]). DEN can be conducted when there is an absolute indication for this treatment option even after two times of drainage-based step-up interventions.

### Criteria for discontinuing or modifying allocated interventions {11b}

The criteria for discontinuing or modifying allocated interventions after the randomisation are as follows:

1. Participants request or withdraw the consent.
2. Participants turn out not to fulfil the eligibility criteria.
3. WON becomes asymptomatic before the allocated interventions are initiated.
4. Participants cannot continue to receive the allocated interventions due to worsened WON, comorbidities, or adverse events.
5. Participants become pregnant.
6. The WONDER-01 trial is terminated.
7. Investigators consider that discontinuation or modification of the allocated interventions is appropriate from the clinical perspective.

### Strategies to improve adherence to interventions {11c}

In the immediate necrosectomy group, it may be difficult to advance a gastroscope thought the LAMS due to inadequate expansion of the stent. In such cases, the LAMS will be dilated using a balloon catheter, potentially facilitating technical success of the allocated treatment.

### Relevant concomitant care permitted or prohibited during the trial {11d}

All relevant concomitant care [33] and interventions can be administered according to the local clinical practice during the trial interventions.

### Provisions for post-trial care {30}

There are no specific provisions for the post-trial care, which will be done according to the local clinical practice.

### Outcomes {12}

Table 1 summarises the primary and secondary outcome measures in the WONDER-01 trial. The primary endpoint is time from randomisation to clinical success. Clinical success is defined as (1) a decrease in a WON size to 3 cm or less and (2) an improvement of at least two out of the three inflammatory markers (i.e. body temperature, white blood cell count, and C-reactive protein). Patients who do not undergo clinical success within 6 months of the randomisation are treated as cases with clinical failure. We will evaluate time to clinical success as the primary endpoint as this outcome measure is correlated with duration of intensive treatment and thus a burden on patients and the health care system. The median time to clinical success will be used to evaluate the overall treatment efficacy of a given intervention. Other outcomes will be summarised as medians (interquartile ranges) or mean ± standard deviations, as appropriate, for continuous variables and number (percentage) of patients for categorical variables.

### Participant timeline {13}

**Fig. 1:**
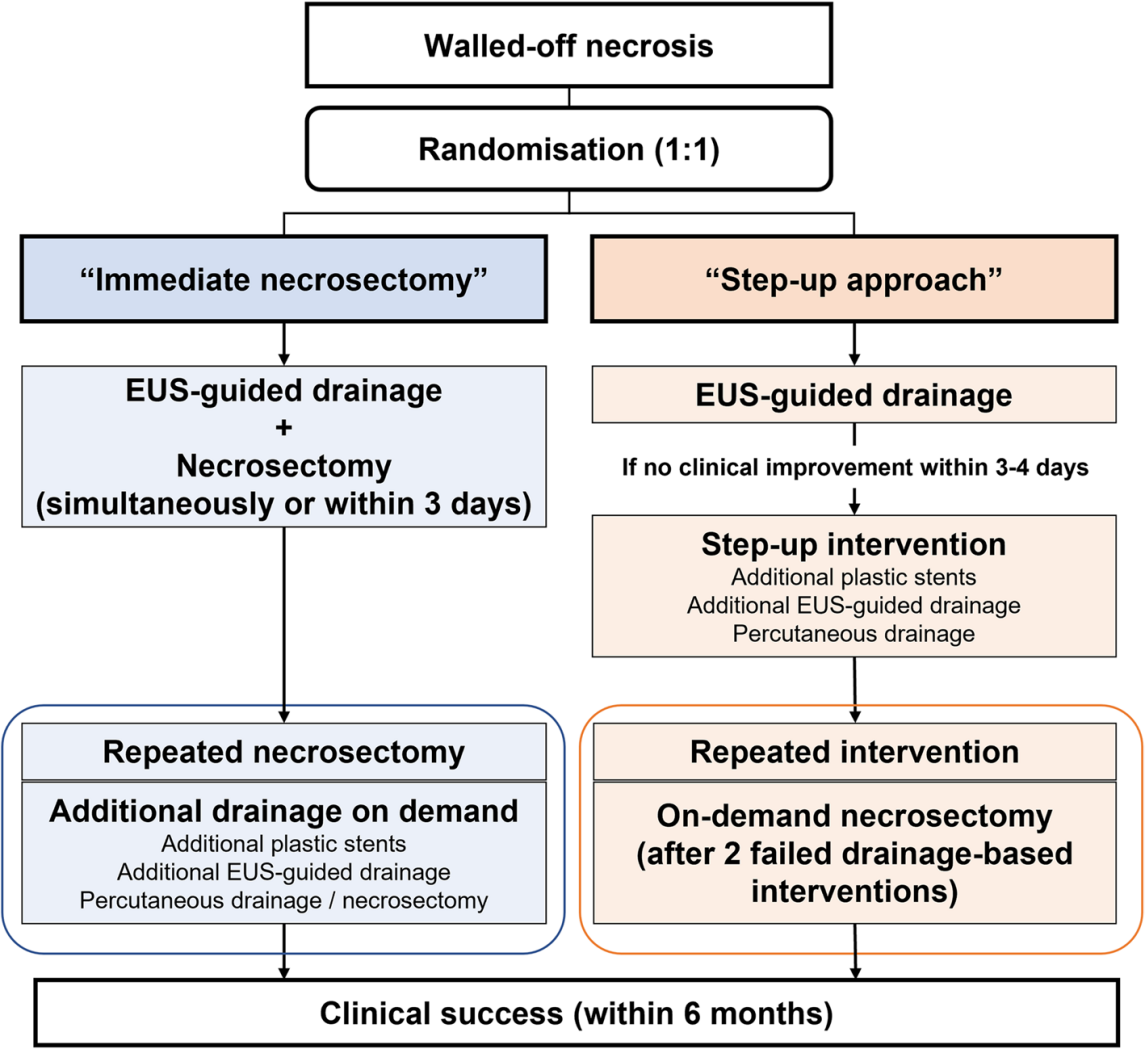
Flow diagram of interventions for the experimental and control groups in the WONDER-01 trial

A schematic diagram of the trial timeline for participants is illustrated in Fig. 1.

### Sample size {14}

For the sample size calculation, we assumed that the rates of clinical success at 60 days of the initial EUS-guided drainage were 60% in the immediate necrosectomy group and 35% in the step-up approach group according to preliminary data from a retrospective analysis by the WONDERFUL study group (under submission). When we planned 36 months for patient accrual and 6 months for follow-up, 64 patients were required with a two-sided α level of 0.05 and a power of 0.80. Taking the dropout into account, we planned a sample size of 70 patients (35 patients per arm).

#### Recruitment

Investigators at each institution will create a list of all patients presenting with WON regardless of requirement of interventional treatment and screen the eligibility for all the patients in the list. The principal investigator will create a webpage to introduce the current trial to hospitals and increase referrals. For cases with equivocal computed tomography (CT) findings in terms of the eligibility, the expert panel consisting of seven gastroenterologists and two board radiologists will hold an online meeting or e-mail communication upon consultation and make a decision within 24 h.

## Assignment of interventions: allocation

### Sequence generation {16a}

Eligible patients with WON will be allocated randomly to either the immediate necrosectomy group (experimental group) or the step-up approach group (control group) based on random sequence generated by the web-based system (University Hospital Medical Information Network Internet Data and Information System for Clinical and Epidemiological Research, cloud version [UMIN INDICE Cloud], https://www.umin.ac.jp/indice/cloud.html). The WONDER-01 trial employs the completely randomised design without blocking or stratification.

### Concealment mechanism {16b}

The web-based randomisation system will be utilised, and therefore, the randomisation process will be concealed completely.

### Implementation {16c}

Investigators will enrol eligible patients and register them to the web-based randomisation system, which will assign the participants to interventions.

## Assignment of interventions: blinding

### Who will be blinded {17a}

Due to the nature of the experimental and control interventions, the participants and investigators will not be blinded to the assigned groups. The outcome evaluators and data analysists will be blinded to the assignment.

### Procedure for unblinding if needed {17b}

n/a. The participants and investigators will not be blinded to the assigned groups.

## Data collection and management

### Plans for assessment and collection of outcomes {18a}

Clinical parameters at baseline and outcome variables have been pre-defined. To promote the quality of data on the primary endpoint (i.e. clinical success), the expert panel will review the clinical course and CT images upon request. Data on those variables will be collected from the electronic medical chart at each centre. The schedule of enrolment, randomisation, interventions, and assessments is summarised in Table 3.

**Table 3:** Schedule of interventions and assessments in the WONDER-01 trial

	Study period					
	Pre-intervention	Intervention (within 6 months of randomisation)				Post-intervention
	≤ 10 days before randomisation		Within 3 days of randomisation	7 days after intervention	Clinical success	After five years of clinical success
	Screening	Randomisation	Intervention		Assessment	Follow-up
Informed consent	X					
Eligibility screening	X					
Assessment of WON	X					
Assessment of symptoms	X				X	
Body temperature	X			X	X	
Blood test^a^	X			X	X	
Imaging study^b^	X			X^c^	X	
Electrocardiogram	X					
Randomisation		X				
Interventions			X			
Assessment of primary endpoint					X	
Assessment of secondary endpoints				X	X	X
Monitoring of adverse events				X	X	X

^a^A blood test includes the following items: white blood cell count, haemoglobin, platelet count, albumin, aspartate aminotransferase, alanine aminotransferase, gamma-glutamyl transpeptidase, alkaline phosphatase, total bilirubin, amylase, lipase, blood urea nitrogen, creatinine, C-reactive protein, and international normalised ratio of prothrombin time

^b^Contrast-enhanced computed tomography is performed unless there are contraindications for contrast use. Magnetic resonance imaging may be performed on the investigator’s discretion

^c^Imaging studies are performed at least every 2–3 weeks during the intervention period

Abbreviation: *WON* Walled-off necrosis

The investigators at each centre will collect relevant patient data from the electronic medical chart and input anonymised data to the trial database. The standardised trial database has been constructed using the Microsoft Access software (Microsoft Corp., Tokyo, Japan) and has been distributed to participating centres. The database file will be uploaded to the online storage that can be accessed only by the investigators.

### Plans to promote participant retention and complete follow-up {18b}

The enrolled patients will undergo all interventions on an inpatient basis and will be requested to visit the outpatient clinic at least once a month after the discharge. When patients do not make a scheduled visit, the investigators will call the patients to follow up on the patients’ conditions and make a subsequent appointment.

### Data management {19}

The investigators will upload collected patient data to the online storage. The data manager will download and integrate the files and then store the integrated database in a password-locked stand-alone computer at the research management office at The University of Tokyo Hospital (Tokyo, Japan). The data manager will also screen for missing or unplausible data and ask the corresponding investigator at each centre for data check. The document of data management procedures has been approved by the institutional review board at The University of Tokyo Hospital.

### Confidentiality {27}

A fake ID number will be assigned to each potential or enrolled participant, and all patient data will be anonymised as soon as they are collected. The corresponding investigator at each centre will store the list matching the fake and hospital ID numbers in a password-locked stand-alone computer.

### Plans for collection, laboratory evaluation and storage of biological specimens for genetic or molecular analysis in this trial/future use {33}

n/a. In the present trial, biospecimens will not be collected for genetic or molecular analyses.

## Statistical methods

### Statistical methods for primary and secondary outcomes {20a}

In the primary analysis, we will compare times to clinical success between the immediate necrosectomy and step-up approach groups. In our primary hypothesis testing, cumulative survival probabilities of times to clinical success will be estimated using the Kaplan–Meier product-limit method and be compared using the log-rank test. Patients are censored at the time-point of salvage surgery, the last follow-up, 6 months of the randomisation, or death, whichever came first. In the secondary analyses, we will use Student’s *t*-test or Wilcoxon rank-sum test, as appropriate, for continuous variables; the chi-square test or Fisher’s exact test, as appropriate, for categorical variables; and the log-rank test for time-to-event variables.

The two-sided α level of 0.05 was used for statistical significance for all analyses. All analyses will be conducted for the intention-to-treat population, and examinations of per-protocol population will represent secondary analyses.

### Interim analyses {21b}

There is no planned interim analysis.

### Methods for additional analyses (e.g. subgroup analyses) {20b}

In subgroup analyses of time to clinical success stratified by clinically relevant parameters (e.g. the size of WON, the proportion of necrotic components in the WON cavity [estimated and classified as < 30%, 30–60%, or > 60% [34], based on preprocedural CT findings], extension status of WON), we will assess statistical heterogeneity in the hazards by a specific variable by evaluating the Wald test on a cross-product of the variable and the treatment group in the Cox proportional hazards regression model. The multivariable Cox regression model will be used to adjust for potential imbalance of confounders and to calculate hazard ratios for clinical success comparing the immediate necrosectomy to the step-up approach.

### Methods in analysis to handle protocol non-adherence and any statistical methods to handle missing data {20c}

In the primary analyses of time to clinical success, patients who lose to follow-up will be treated as censored cases at the time of the last follow-up. In multivariable Cox regression models, we will assign a major category for missing data on categorical covariates and a mean or median value, as appropriate, for missing data on continuous covariates. We will confirm that excluding cases with missing data does not alter our findings substantially. In the analyses of the secondary endpoints, patients with missing data on the corresponding variable will be excluded.

### Plans to give access to the full protocol, participant-level data and statistical code {31c}

The full protocol and statistical code will be accessible to the public on reasonable request. There is no plan of granting public access to participant-level dataset. The results of the present trial will be presented at conferences/seminars and be published in a peer-reviewed journal to maximise the chances of dissemination of the results to the public. The results will also be posted in the trial registries, ClinicalTrials.gov, University Hospital Medical Information Network (UMIN), and Japan Registry of Clinical Trials (jRCT).

## Oversight and monitoring

### Composition of the coordinating centre and trial steering committee

The trial steering committee consists of the principal investigator (Y.N.), co-principal investigators (H.I. and I.Y.), and the representative of the investigator team at each centre. The committee will hold an online meeting every 2–3 months to check the progress of the trial and share the information on severe adverse events (SAEs). The Clinical Research Support Centre at The University of Tokyo Hospital provides day-to-day organisational support for the trial which monitors an annual report of the trial progress and SAEs submitted by the principal investigator.

### Composition of the data monitoring committee, its role and reporting structure {21a}

The Data Monitoring Committee has a monitoring manager who independently oversees the progress of the trial and compliance with the protocol using pre-defined monitoring forms. The monitoring manager will check the information in the electronic medical charts and the trial database to confirm the appropriateness of enrolment, allocation, interventions, and follow-up as well as missing data for outcome evaluation. The monitoring manager will report to the principal investigator whether there is a deviation from the protocol. The monitoring will be done independent from the competing interests.

### Adverse event reporting and harms {22}

SAEs are defined as unfavourable events that cause patient death, life-threatening events, unexpected or prolonged hospitalisation, or permanent or severe disability, regardless of plausibility of causal associations with the trial interventions. All SAEs will be managed by treating investigators at each centre. Consulting with the treating investigators, the principal investigator will evaluate the plausibility of the causal association using the MEDDEV (MEDical DEVices Documents) guidelines 2.7/1 revision 4, proposed by European Commission [35]. In the case of SAEs, the investigators will submit a report to the principal investigator using a pre-defined form. Subsequently, the principal investigator will consult with the institutional review board and the director of The University of Tokyo Hospital for the continuation of the trial. The information on SAEs will be shared with participating investigators to ensure the safety of the trial interventions.

### Frequency and plans for auditing trial conduct {23}

No audit is planned in the present trial. The data will be monitored by the committee.

### Plans for communicating important protocol amendments to relevant parties (e.g. trial participants, ethical committees) {25}

We will submit any modifications of the protocol (e.g. changes to the eligibility criteria, participating centres, endpoints, analyses) to the institutional review board at each centre and obtain the approval. The information at the trial registries will be updated accordingly. Trial participants will be informed about the amendments approved by the institutional review boards.

### Dissemination plans {31a}

The results of the present trial will be presented at conferences/seminars and be published in a peer-reviewed journal to maximise chances of dissemination of the results to healthcare professionals and the public and to contribute to the improvement of public health. The results will also be posted in the trial registries, ClinicalTrials.gov, UMIN, and Japan Registry of Clinical Trials (jRCT). Plain language summaries of the findings will be shared with trial participants on request.

## Discussion

WONDER-01 has been designed as a multicentre RCT that aims to evaluate the efficacy and safety of the immediate DEN approach compared to the step-up approach in patients with WON receiving EUS-guided drainage. Our survey of expertise endoscopists demonstrated considerable heterogeneity in clinical practice of endoscopic treatment of WON, suggesting an urgent need for RCTs for standardisation of the treatment protocol in this setting. The promise of clinical effectiveness of immediate DEN following EUS-guided drainage of WON has been supported only by retrospective series. In a multicentre comparative study, DEN initiated during the same session of EUS-guided drainage appeared to associate with a smaller number of DEN without no substantial increase in the risk of adverse events [14]. These lines of evidence prompted us to design a RCT to investigate the potential of immediate necrosectomy in managing endoscopically treated WON effectively.

The WONDER-01 trial has strengths in addition to those of RCTs in general. First, enrolment of participants at multiple centres will likely ensure the generalisability of our findings; this strength is considerably important given the variations in clinical practice of adjunctive and supportive treatment during the periprocedural period of EUS-guided treatment of WON [19, 33]. In addition, the relative rarity of WON patients requiring interventions may hamper the timely enrolment of participants. To encourage the enrolment, the current trial will be conducted at 23 centres (as of November 2022), and additional centres will be recruited if required. Therefore, the trial will be completed within the planned accrual period if 2 to 3 participants are enrolled at each centre (approximately one participant annually). Second, the broad inclusion criteria have been set to ensure the representativeness of our participants as patients with large-size WON. Third, EUS-guided drainage of WON and subsequent interventions including DEN will be performed on an inpatient basis according to the local practice. The hospitalisation will allow us to evaluate adverse events accurately during the periprocedural period.

We acknowledge potential challenges of the current trial. First, there may be difficulties in accurately differentiating WON from other types of fluid collections such as pseudocysts. Nonetheless, we have set up the online meeting platform so that we can hold the expert panel consisting of multiple gastroenterologists and board radiologists and draw a mature conclusion on the eligibility in a timely fashion. Second, due to the nature of the interventions in the experimental and control groups, the participants and endoscopists cannot be blinded to the assigned groups. Yet our outcome evaluators and data analysists will be blinded to the assignment to mitigate a bias due to the open-label procedures.

The current clinical guidelines have no recommendation on the timing of DEN following EUS-guided drainage of WON since no clinical RCTs have examined the timing of DEN in this setting [4, 6, 36]. Therefore, the results of this large, multicentre RCT are expected to add to the literature and help us to implement evidence-based practice for better clinical outcomes of patients with WON.

## Trial status

The current version of the protocol is 6.0, which has been updated on 28 September 2022. The recruitment started on 29 July 2022 and is scheduled to be completed on 1 April 2025.

## Data Availability

All investigators participating in the present trial will have access to the final study dataset. The anonymised study data and statistical methods can be shared from the primary investigator (Y.N.) on reasonable request, but appropriate approval at the institutional review board may be required.

## Abbreviations

CT: Computed tomography
DEN: Direct endoscopic necrosectomy
EUS: Endoscopic ultrasound
LAMS: Lumen-apposing metal stent
PFC: Pancreatic fluid collection
RCT: Randomised controlled trial
SAE: Severe adverse event
WON: Walled-off necrosis
